# Recent progress in neuromorphic and memory devices based on graphdiyne

**DOI:** 10.1080/14686996.2023.2196240

**Published:** 2023-04-14

**Authors:** Zhi-Cheng Zhang, Xu-Dong Chen, Tong-Bu Lu

**Affiliations:** a The Key Laboratory of Weak Light Nonlinear Photonics, Ministry of Education, School of Physics, Nankai University, Tianjin, China; b MOE International Joint Laboratory of Materials Microstructure, Institute for New Energy Materials and Low Carbon Technologies, School of Material Science and Engineering, Tianjin University of Technology, Tianjin, China

**Keywords:** Graphdiyne, optoelectronic memories, ultrafast nonvolatile memories, artificial synapses, neuromorphic computing, artificial visual systems

## Abstract

Graphdiyne (GDY) is an emerging two-dimensional carbon allotrope featuring a direct bandgap and fascinating physical and chemical properties, and it has demonstrated its promising potential in applications of catalysis, energy conversion and storage, electrical/optoelectronic devices, etc. In particular, the recent breakthrough in the synthesis of large-area, high-quality and ultrathin GDY films provides a feasible approach to developing high-performance electrical devices based on GDY. Recently, various GDY-based electrical and optoelectronic devices including multibit optoelectronic memories, ultrafast nonvolatile memories, artificial synapses and memristors have been proposed, in which GDY plays a crucial role. It is essential to summarize the recent breakthrough of GDY in device applications as a guidance, especially considering that the existing GDY-related reviews mainly focus on the applications in catalysis and energy-related fields. Herein, we review GDY-based novel memory and neuromorphic devices and their applications in neuromorphic computing and artificial visual systems. This review will provide an insight into the design and preparation of GDY-based devices and broaden the application fields of GDY.

## GRAPHICAL ABSTRACT

**Figure:**
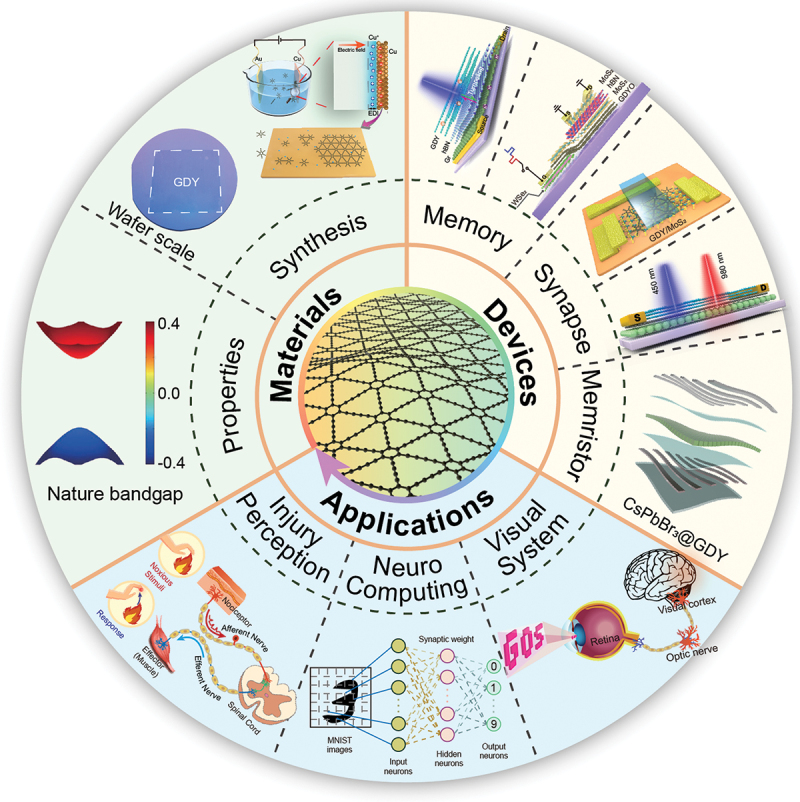

## 1. Introduction

In the era of artificial intelligence (AI), big data and Internet of Things (IoTs), huge amounts of data are created annually, resulting in the explosive growth of demand for high-performance memory and processing devices [1–3]. The improvement of computing capacity and storage capacity often depends on the degree of integration of the chip. It is impossible to achieve a ‘qualitative leap’ to scale down the physical size of the chip unit by relying solely on Moore’s law due to its physical limitation [4,5]. In addition, the traditional von Neumann computer has spatially separated storage and processing units, and the time delay and loss of data transmission greatly reduce the computing speed and energy efficiency, resulting in the mismatch of memory and processor (i.e. memory wall) [6–16]. Limited by the exhaustion of Moore’s Law and the von Neumann bottleneck [7,17,18], the traditional complementary metal oxide semiconductor (CMOS) integration in the von Neumann computing system is unable to meet the urgent demand of hardware computing power improvement.

The human brain can process synaptic events obtained from external stimuli in a highly parallel manner, with less power consumption than the von Neumann computing system [19–21]. Inspired by the human brain, neuromorphic devices that integrate sensing-memory or sensing-memory-computing, featuring extremely high parallelism and ultra-low power consumption, are regarded as the most promising approaches to overcome the limitations of traditional computing architecture [22–31]. In the past decade, great advances have been made in neuromorphic devices, including artificial synapses and neurons [32–41]. For example, optoelectronic synaptic devices integrate optical signal sensing and synaptic functions into one device [7,11,42–57], and their simple architecture and highly integrated functionalities are considered as the highly promising neuromorphic devices. In addition, the latest researches show that some novel memories (e.g. multi-bit optoelectronic memory [58] and high-speed nonvolatile memory [59–61]) and memristors also provide a new strategy for realizing high-capacity and efficient storage, providing a possible way to fill the huge performance gap between memories and processors.

Two-dimensional (2D) materials have attracted numerous attentions due to their unique properties, which provide promising candidates for the development of neuromorphic devices. The atomic thickness and atomically sharp interface without surface dangling bonds facilitate the stacking of arbitrary 2D materials to construct van der Waals heterostructures with rich properties [15]. Up to now, the preparation techniques of wafer-scale 2D materials, for example, graphene [62,63], hexagonal boron nitride (hBN) [64], and MoS_2_ [65], are gradually mature, and 2D materials also have great compatibility with mainstream silicon-based complementary metal-oxide-semiconductor (CMOS) technologies [66], facilitating the fabrication of large-scale integrated circuits based on 2D materials. Graphdiyne (GDY), an emerging two-dimensional (2D) carbon allotrope material, has fascinating physical and chemical properties due to its highly π-conjugated structure and abundant electrons [67–72], demonstrating extensive potential applications in catalysis [73–84], biomedicine [85–91], energy storage [92–94], etc. In addition, GDY has a layer-dependent natural band gap and theoretically highly carrier mobility [95–98], and thus its applications in electronic and optoelectronic devices have attracted tremendous attentions. With the recent breakthroughs in the synthesis of large-area and high-quality GDY ultrathin films [99–102], a variety of GDY-based novel devices have been developed in recent years, such as artificial synapses [103–106], memories [100,107–109], memristors [110,111] and photodetectors [112–114]. In these devices, GDY always acts as a key functional layer that absorbs light and traps charge carriers, which is crucial for device performance. These results demonstrate that GDY holds considerable promise for advancing the next-generation high-speed low-energy electronic/optoelectronic devices.

**Figure 1.**
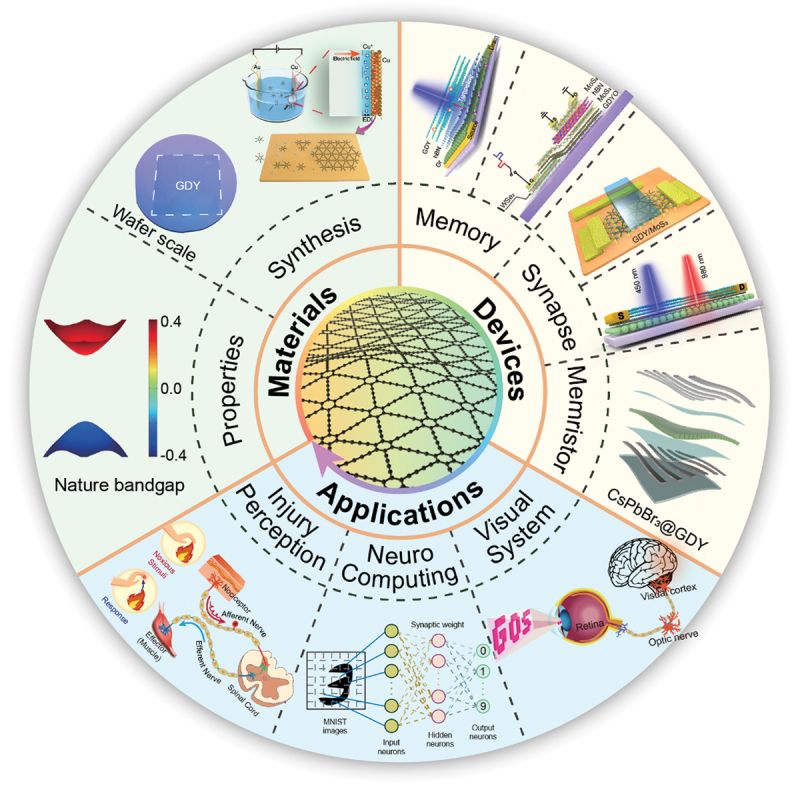
An overview of GDY used for novel electrical/optoelectronic devices and neuromorphic applications. Reprinted from [115] with permission; © 2013, Royal Society of Chemistry, UK. Reprinted from [103] with permission; © 2021, Springer. Reprinted from [100] with permission; © 2021, Elsevier Inc. Reprinted from [109] with permission; © 2022, Springer Nature Ltd. Reprinted from [105] with permission; © 2021 Wiley-VCH, Weinheim, Germany. Reprinted from [104] with permission; © 2020, American Chemical Society. Reprinted from [111] with permission; © 2022, Tianjin University and John Wiley & Sons Australia, Ltd.

Here, we review the recent advances of GDY-based devices including optoelectronic memories, ultrafast nonvolatile memories, artificial synapses and memristors, and their applications in neuromorphic computing, artificial visual systems, nociceptive signal processing, associative learning and logic-in-memory (Figure 1). This review will provide guidance for the development of novel neuromorphic devices based on GDY.

## 2. GDY-based neuromorphic devices

In the human brain, the computation and processing of various information associative learning, forgetting, and decision-making rely on neural networks. As the key components of neural networks, biological synapses carry the exchange, transmission and processing of information [116]. Generally, each synapse is composed of the presynaptic terminal, postsynaptic terminal, and synaptic cleft, which allow presynaptic neurons to transmit chemical signals to postsynaptic neurons through neurotransmitters [117,118]. Through the interconnection between a large number of synapses and neurons, humans can realize different functions, such as touch, vision, hearing, and emotions. Based on the highly parallel processing system, biological neural networks can process synaptic events in a high-speed and low-energy manner.

Inspired by the human brain, neuromorphic devices, including artificial synapses and neurons, have been proposed to implement neuromorphic computing. Especially, artificial synapses inspired by biological synapses have attracted great attentions in recent years. Various synaptic devices have been proposed with a structure of two-terminal memristors and three-terminal transistors [7,11,42–57]. 2D materials and heterostructures present great advantages for the development of neuromorphic devices due to their atomical thickness, atomically sharp interface and free of surface dangling bonds [15]. Especially, the excellent physical and chemical properties of GDY, including the ordered pore structure, direct bandgap, broadband light absorption, strong charge trapping, low ion diffusion barrier, and excellent flexibility, are favorable for applications in neuromorphic devices. For instance, the abundant defects in GDY introduced during the synthesis process endow GDY with great potential in neuromorphic devices since the long-term trapping and slow release of charges at these charge-trapping sites facilitate the emulation of various synaptic plasticity [119]. The density of defects can also be controlled by defect engineering and post-treatment. In addition, the strong and broadband absorption of GDY make it a promising candidate in optoelectronic synapses, in which the photo-generated charges can be effectively trapped in GDY to emulate the synaptic response. We believe all these features are crucial for applications of GDY in neuromorphic devices.

### 2.1 Optoelectronic synapse

Optoelectronic synapses that integrate optical signal detection and synaptic function in a single device have shown promising prospects in neuromorphic visual systems. Artificial neural networks (ANNs) constructed by optoelectronic synapses can significantly improve computing and energy efficiency [52]. In addition, optoelectronic synapses feature advantages of wide bandwidth, low crosstalk, low power loss and negligible resistance-capacitance delay [37,120]. Hence, optoelectronic synapses have been widely investigated in recent years.

**Figure 2.**
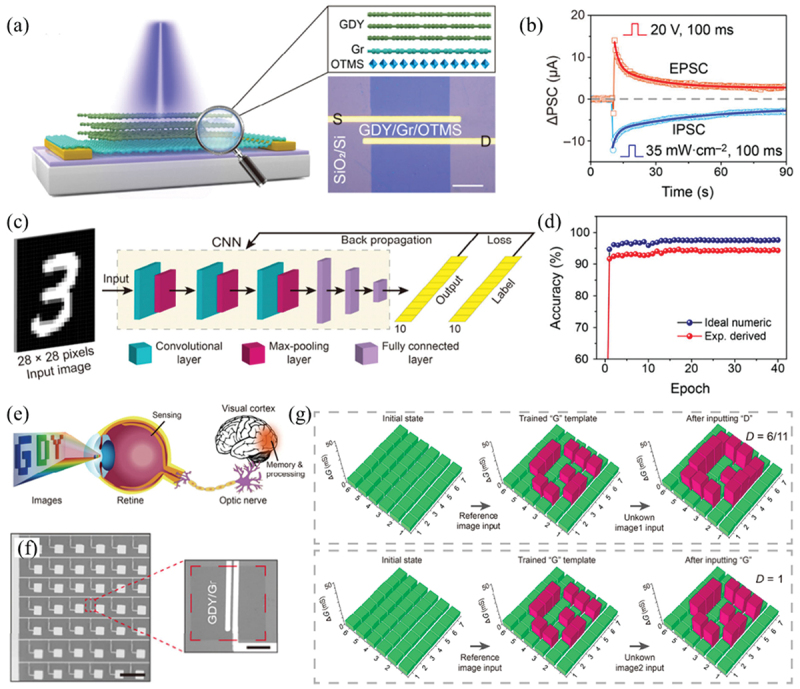
Optoelectronic synapses based on GDY van der Waals heterostructures. (a) Schematic illustration (left) and optical image (right) of an optoelectronic synapse based on GDY/graphene heterostructure. (b) EPSC and IPSC triggered by a *V*_bg_ pulse (20 V, 100 ms) and optical pulse (450 nm, 35 mW cm^−2^, 100 ms), respectively. (c) Schematic of a CNN for recognition of handwritten digits with 28 × 28 pixels. (d) Recognition accuracy evolution with training epochs for 28 × 28 pixels handwritten digit image without noise. (e) Schematic of the human visual system for image sensing, memory and processing. (b) Scanning electron microscopy (SEM) images of a GDY/Gr synapse array with 7 × 6 panels. Scale bars, 300 μm and 25 μm, respectively. (g) Distinction between the reference image ‘G’ and the unknown images 1 (‘D’) and 2 (‘G’), respectively. Reprinted from [103] with permission; © 2021, Tsinghua University Press and Springer-Verlag GmbH Germany, part of Springer Nature.

Chen and co-workers developed an optoelectronic synapse based on GDY/graphene vertical heterostructure prepared by van der Waals epitaxy (Figure 2(a)), in which GDY is used as the photosensitive charge trapping layer and graphene is used as the conductive channel [103]. The excitatory and inhibitory synaptic responses of the device are achieved by applying gate-voltage pulse and optical stimulus, respectively (Figure 2(b)). When applying a positive voltage pulse to the gate terminal, the Fermi level of graphene is elevated, resulting in the injection of electrons from graphene to GDY. These injected electrons are trapped in GDY, increasing the hole concentration in graphene channel. In turn, numerous electron–hole pairs are generated in GDY under illumination, and photogenerated electrons are transferred into graphene driven by the built-in electric field at the GDY/graphene interface, leaving photogenerated holes trapped in GDY. As a result, the hole concentration in graphene decreases via photo-gating effect, and thus the conductance of graphene channel decreases. Various synaptic functions, such as short-term plasticity (STP), long-term plasticity (LTP), paired pulse facilitation (PPF), spike-rating-dependent plasticity (SRDP) and associative learning, have been emulated by this device. Moreover, the near-linear and symmetric conductance updating behaviors of the device enable its application in neuromorphic computing. As a demonstration, the classification of handwritten digits was performed using a convolutional neural network (CNN) with the parameters of this device (Figure 2(c)). As shown in Figure 2(d), a recognition accuracy of 94.3% is achieved is several epochs for 28 × 28 pixels handwritten digits from the modified U.S. National Institute of Standards and Technology (MNIST) dataset, which is slightly lower than the ideal numeric simulation result (97.5%). In addition, an artificial visual system with 7 × 6 pixels was constructed, which can realize real-time detection, in situ memory and image differentiation (Figure 2(e–g)).

Restricted by the unidirection of the photoresponse, most of the reported optoelectronic synapses require an opto-electrically mixed modulation, for example, a gate pulse for excitatory synaptic response and an optical stimulation for inhibitory response, to achieve bidirectional conductance [55,120–122]. However, the introduction of electrical stimulation inevitably offsets the advantages introduced by light stimulation, such as the wide bandwidth, low crosstalk, low power loss and negligible resistance-capacitance delay. It is urgent to develop fully light-controlled synaptic devices that both excitatory and inhibitory synaptic behaviors can be implemented in an optical pathway.

**Figure 3.**
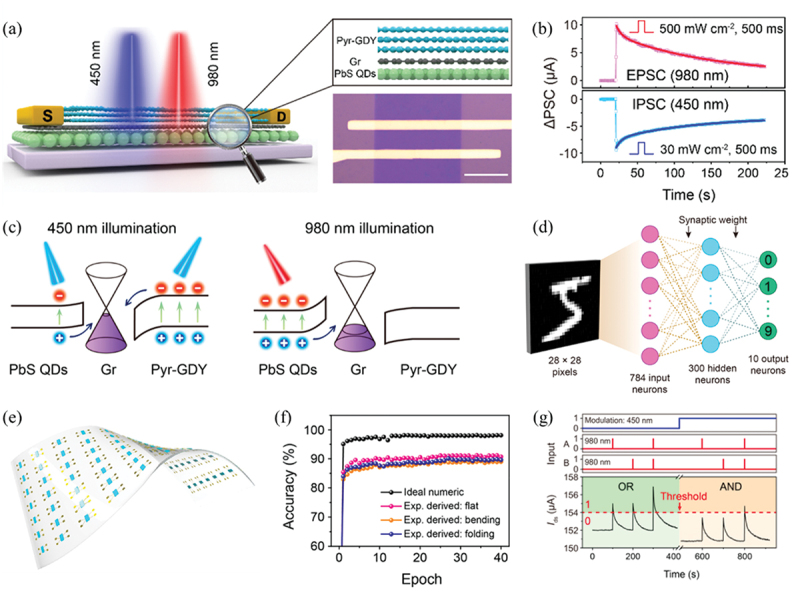
Optoelectronic synapses based on PbS QDs/GDY/Gr van der Waals heterostructure. (a) Schematic illustration (left) and optical image (right) of a fully light-controlled synapse with a structure of Pyr-GDY/graphene/PbS QDs. (b) EPSC and IPSC of the device triggered by an optical pulse with wavelengths of 980 and 450 nm, respectively. (c) Energy band alignment for the Pyr-GDY/Gr/PbS heterostructure under 450 nm illumination and 980 nm illumination. (d) Schematic of a three-layer neural network for recognition of handwritten digits with 28 × 28 pixels. (e) Schematic of the optical synapse array fabricated on a flexible PET substrate with thickness of 0.1 mm. (f) Recognition accuracy evolution with training epochs for 28 × 28 pixels handwritten digit images without noise. (g) ‘OR’ and ‘AND’ logic functions realized by using binary 980 nm signals as inputs a and B, and using 450 nm illumination as modulatory input. Reprinted from [104] with permission; © 2020, American Chemical Society.

Chen and co-workers constructed a fully light-controlled synaptic device based on Pyr-GDY/graphene/PbS quantum dots (QDs) vertical heterostructure (Figure 3(a)) [104], where Pyr stands for pyrenyl, a radical of pyrene. Benefitting from the different absorption peaks of Pyr-GDY and PbS QDs and the well-engineered band bending at the Pyr-GDY/graphene and graphene/PbS QDs interfaces, this synaptic device can emulate both excitatory and inhibitory postsynaptic current (EPSC/IPSC) in an optical pathway (Figure 3(b)). As shown in Figure 3(c), Pyr-GDY is the main photoresponsive layer when the device is illuminated by a 450 nm light, and photogenerated electrons are transferred from Pyr-GDY to graphene channel driven by the built-in electric field between Pyr-GDY and graphene, leaving holes trapped in Pyr-GDY layer. Since the graphene channel here is hole-dominated carrier, the positive photogating effect induced by the trapped holes in Pyr-GDY would induce a decrease of channel conductance, i.e. IPSC. On the contrary, when the device is illuminated by a 980 nm light, which is mainly absorbed by the PbS QDs layer, the photogenerated holes are transferred from PbS QDs to graphene, and electrons are trapped in PbS QDs, which would induce an increase of channel conductance via photogating effect. In addition, this device has successfully emulated PPF, SRDP, LTP/LTD and associative learning in a fully optical pathway. This device also exhibits excellent flexibility, and comparable conductance updating behaviors are achieved at different bending states. A three-layer ANN was constructed based on the parameters of this device at different bending states, and the recognition accuracies of the networks for handwritten digits are 90.8% (flat), 89.5 (folding) and 88.9% (bending), respectively (Figure 3(d–f)). Furthermore, the bidirectional optical response of the device enables its application in optical logic operations [123]. Logic functions of ‘AND’, ‘OR’, ‘NAND’, ‘NOR’ and ‘XOR’ are implemented in an optical pathway by adjusting the modulation and input pulses (Figure 3(g)).

### 2.2 GDY-based electrolyte-gated synaptic transistor

Electrolyte-gated transistors (EGTs) using ions in electrolyte to modulate channel conductance have been widely investigated owing to their prominent analog switching behaviors similar to biological synapses [17]. Driven by a small gate bias, ions in electrolyte accumulate near the surface of the channel and modulate the channel conductance via EDL gating effect [124,125], whereas ions can penetrate into the channel under a high gate bias and change the channel conductance via electrochemical doping or redox mechanisms [16,126,127]. However, EDL-based EGTs suffer poor retention characteristics since the accumulated ions diffuse back to bulk electrolyte after gate bias, while a relatively large voltage is required for EGTs based on electrochemical doping and redox, and frequent intercalation/deintercalation of ions into a 2D semiconductor channel might damage the channel lattice and thus leads to a poor endurance [16].

**Figure 4.**
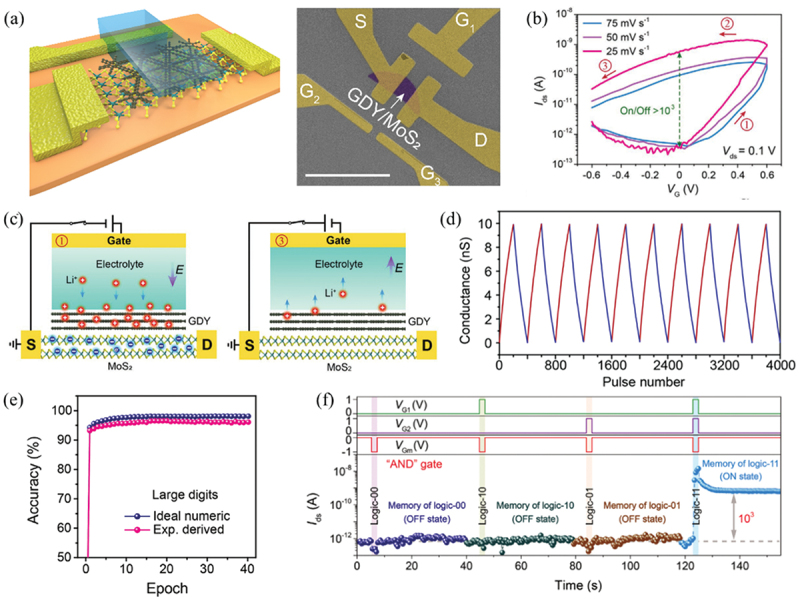
Electrolyte-gated synapse device based on GDY/MoS_2_ heterostructure. (a) Schematic illustration (left) and pseudo-color scanning electron microscopy (SEM) image (right) of an EGT based on GDY/MoS_2_ heterostructure. (b) Transfer curve of the EGT with side-gate voltage sweeping from −0.6 to 0.6 V and back to −0.6 V with different sweeping rate. (c) the intercalation and ejection processes of Li ions to GDY layers, corresponding to the programming and erasing processes. (d) Cyclic conductance-updating with 200 discrete states induced by ±1 pA 10 ms pulses between source and gate. (e) Recognition accuracy evolution with training epochs for large handwritten digits. (f) Output signals for the processing of logic ‘AND’ gates with in situ memorization. Reprinted from [105] with permission; © 2021, Wiley-VCH.

In order to obtain an EGT combining advantages of low operation voltage, large dynamic range, long retention, low energy consumption and high robustness, Chen and co-workers proposed a GDY/MoS_2_-based EGT using MoS_2_ as channel and GDY as Li-ion-storage layer (Figure 4(a)) [105]. GDY has demonstrated its potential in Li-ion storage due to its high Li-ion storage capability (LiC_3_) and low diffusion barrier [128]. The ordered pore structure of GDY facilitates the intercalation of Li^+^ under a small gate bias. A 0.5 V *V*_G_ pulse was used to drive Li^+^ to intercalate into the GDY layer, while a much larger voltage (>3 V) was required for the intercalation of Li^+^ into other 2D materials [16]. In addition, the size of the pores is tunable by designing suitable monomer molecules, and thus this special ion-storage layer can also be used for other ions with larger or smaller sizes. In addition, the evenly distributed large pores of GDY facilitate the injection and extraction of Li ions under a small gate bias. A counterclockwise hysteresis with an on/off ration of 10^3^ was achieved by this device with *V*_g_ sweeping in the range of ±0.6 V, indicating a low operation voltage and large dynamic range (Figure 4(b)). As illustrated in Figure 4(c), Li ions were injected into the GDY layer driven by 0.5 V gate bias and captured in GDY even after removing the bias, resulting in long retention characteristics. Noteworthily, Li ions in electrolyte cannot penetrate into MoS_2_ channel under such a small voltage, which was demonstrated by the electron energy loss spectroscopy (EELS) mapping. Thus, the channel conductance was modulated by the trapped Li ions via the EDL gating effect. Various synaptic behaviors such as EPSC/IPSC, PPF and spike-timing-dependent plasticity (STDP) were also emulated by this GDY-based EGT. This device featured an ultralow energy consumption of 50 aJ μm^−2^, which was orders of magnitude lower than that of other EGTs. In addition, linear and symmetric weight updating with high dynamic range (>10^3^) was also demonstrated (Figure 4(d)), which are important for high-accuracy neuromorphic computing. The recognition accuracy of ANN with the parameters of this EGT for handwritten digits is as high as 96% (Figure 4(e)). Furthermore, the logic-in-memory function has also been implemented by this GDY-based EGT. As a demonstration, Figure 4(f) illustrates the processing of logic ‘AND’ with in-situ memorization. Other logic gates such as ‘OR’, ‘NAND’ and ‘NOR’ with simultaneous storage were also demonstrated.

**Figure 5.**
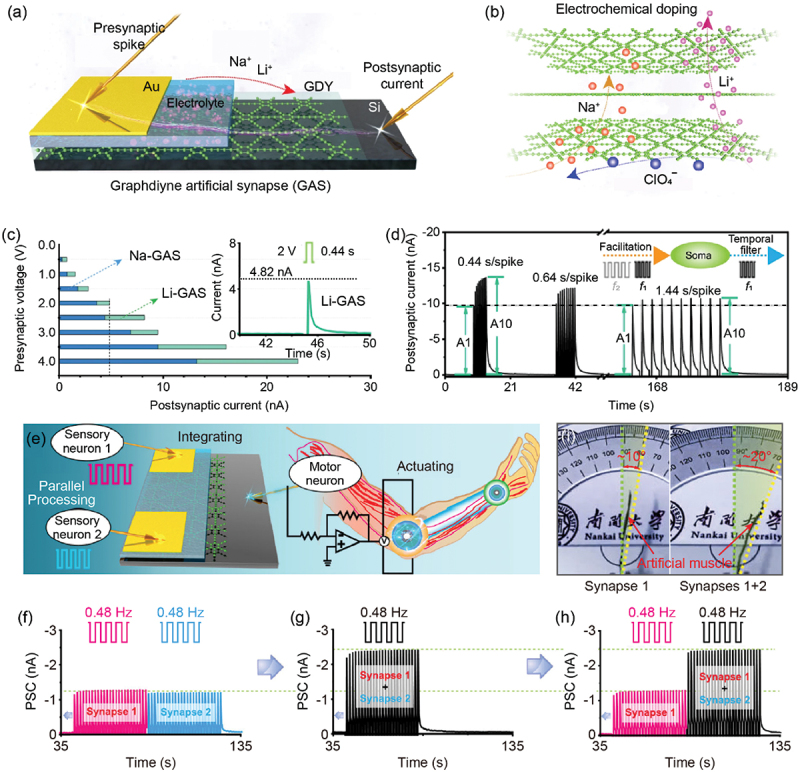
GDY-based artificial synapse (GAS) with an electrolyte/GDY junction. (a) Schematic illustration of GAS. (b) Dynamic diffusion of Li^+^ and Na^+^ between GDY layers. (c) Peak values of the postsynaptic current (PSC) triggered by different amplitudes of positive voltage pulses in Li-GAS (green) and Na-GAS (blue), respectively. Inset: the PSC response triggered by a 2 V/0.44 s voltage pulse in Li-GAS. (d) PSC triggered by 10 successive voltage pulses with different frequencies in Li-GAS. (e) Schematic illustration of an artificial efferent nerve consisting of GAS and artificial muscle. (f–h) PSC of the GAS triggered by two presynaptic input signals (0.48 Hz) with different time periods. (i) Photographs of the artificial muscle flexion under 0.8 Hz pulse sequences from one (left panel) and two (right panel) synapses. Reprinted from [106] with permission; © 2021, Springer Nature Ltd.

Xu and co-workers developed a two-terminal junction-type artificial synapse with a structure of Au/electrolyte/GDY/Si as illustrated in Figure 5(a) [106,129]. While a positive impulse with amplitude of several millivolts was applied to the top Au electrode, alkali metal ions (Li^+^ and Na^+^) were driven to migrate into GDY, leaving anions (ClO_4_–) accumulated at the interface of electrolyte/GDY (Figure 5(b)). The conductance of GDY was enhanced by electrochemical doping of injected alkali metal ions. Since the Li ion is smaller than the Na ion, more Li ions were injected into the GDY layer, resulting in a larger PSC (Figure 5(c)). Since the cations in GDY and anions accumulated at the interface formed an internal electric field, which can extract the injected alkali metal ions from GDY, this device featured a volatile retention characteristic to emulate short-term plasticity (STP). The STP characteristic enabled the device to achieve high-pass filtering (Figure 5(d)). This device also demonstrated its potential in parallelly processing signals transmitted from multiple pre-neurons. As illustrated in Figure 5(e), an artificial efferent nerve that can real-time process parallel information from multiple sensory neurons and trigger the response of actuator was constructed. Impulse stimuli with different frequencies were applied to the artificial efferent nerve as spiking signals from receptors. Figure 5(f–h)) depicts the output signals of this artificial efferent nerve triggered by two impulse stimuli at 0.48 Hz with different time periods. As a result, the cooperation of two sets of input signals can drive a larger bending of the artificial muscle in comparison with that induced by a single input (Figure 5(i)).

## 3. GDY-based memristors

Memristors with a two-terminal cross-bar architecture are regarded as the most promising candidates for hardware implementation of neuromorphic computing and in-memory computing [34,42]. According retention characteristics, memristors can be classified as nonvolatile resistive-switching memristor (RSM) and volatile threshold-switching memristor (TSM), which can be used to mimic synapses and neurons, respectively [34,130,131]. However, most of the existing memristors can operate only in one mode, resistive switching (RS) or threshold switching (TS) modes, and dual-mode memristors with freely switchable operation mode are seldomly reported.

**Figure 6.**
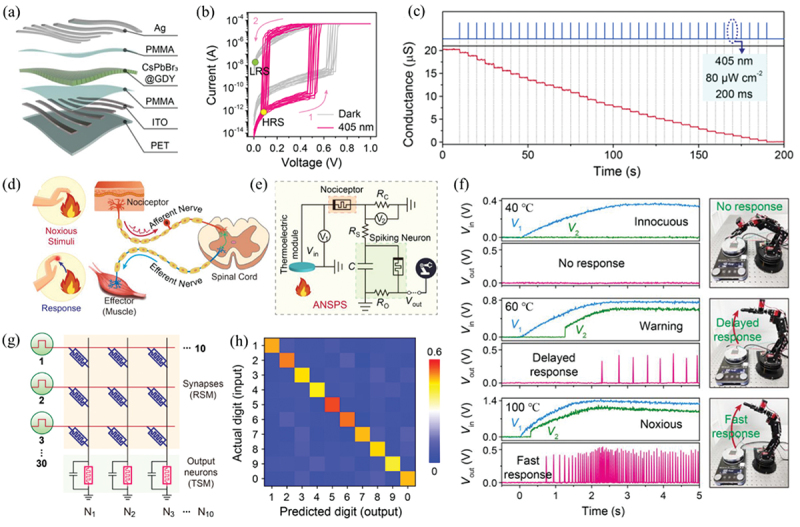
Memristors based on CsPbBr_3_@GDY. (a) Schematic illustration (left) and cross-section scanning electron microscopy (SEM) image (right) of a memristor with a structure of Ag/PMMA/CsPbBr_3_@GDY/PMMA/ITO, where ITO stands for indium tin oxide. (b) *I*–*V* curves of the memristor under 405 nm light illumination, demonstrating the transition from RS mode to TS mode. (c) Response of the memristor triggered by successive optical pulses (405 nm, 20 μW cm^−2^, 100 ms). (d) Schematic of nociceptive signal processing in biological nervous system. (e) Illustration of an artificial nociceptive signal processing system (ANSPS) consisting of a sensor, a nociceptor and a spiking neuron. (f) Response of the ANSPS to innocuous stimulation (40°C, upper panel), warning stimulation (60°C, middle panel) and noxious stimulation (100°C, lower panel), respectively. (g) Illustration of the fully memristive spiking neural network (SNN) based on dual-mode memristor arrays in RS and TS modes for synapses and output neurons, respectively. (h) Confusion matrix between actual (input) and predicted (output) digits. Large values along the diagonal line demonstrate the successful recognition of digits by the fully memristive SNN. Reprinted from [111] with permission; © 2022, Tianjin University and John Wiley & Sons Australia, Ltd.

Chen and co-workers developed an optically controlled dual-mode memristor based on core–shell CsPbBr_3_@GDY QDs [111]. The structure of the device is illustrated in Figure 6(a), in which a close-packed CsPbBr_3_@GDY layer was sandwiched between two poly(methyl methacrylate) (PMMA) layers. As shown in Figure 6(b), this memristor featured a typical RS behavior in dark conduction, and it spontaneously switched to TS mode while under illumination. The mechanism for the RS to TS mode transition can be explained by the dissolution of Ag CFs under illumination. The core–shell CsPbBr_3_@GDY formed a type-II heterojunction, and photogenerated holes were accumulated at the GDY layer driven by the built-in electric field. These holes were transferred to neighboring Ag CFs and oxidized Ag to Ag^+^, resulting in the dissolution of Ag CFs and the spontaneous switching from LRS to HRS [130]. By controlling the intensity and duration of applied light pulse, Ag CFs were partially dissolved by photogenerated holes and over 38 distinct conductive states were obtained by applying successive light pulses (Figure 6(c)), demonstrating great potential in analog computation and multibit storage. This dual-mode memristor has demonstrated its potential in fully memristive nociceptive signal processing system and ANN. As illustrated in Figure 6(d,e), a nociceptive signal processing system was constructed, using this CsPbBr_3_@GDY-based memristor in TS mode to emulate both the nociceptor and spiking neuron. Key features of biological nociceptors, including ‘threshold’, ‘no-adaption’, ‘relaxation’, ‘sensitization’ and the ‘cure’ process, were successfully emulated. As a demonstration, this system was used to perceive external heat stimulation. Figure 6(f) depicts the output spiking signals of the system and corresponding response of robotic arm for different temperature stimuli. This nociceptive signal processing system successfully emulated the responses of humans to different temperatures. A fully memristive ANN was also constructed using RS-mode and TS-mode memristors as artificial synapses and output neurons, respectively (Figure 6(g)). Unsupervised learning for the recognition of digits was performed as a demonstration, and the input digits and output signals presented a one-to-one correspondence as a diagonal matrix (Figure 6(h)), demonstrating the successful recognition of digits.

GDY has demonstrated its potential in various neuromorphic devices as summarized above, in which GDY plays a crucial role for the high performance of these devices. For example, the charge-trapping capability and broadband absorption of GDY is essential for the development of optoelectronic synapses, and the well-designed band structure facilitates the separation of photogenerated charges and the realization of dual-mode memristors. Especially, the ordered-pore structure and excellent Li-ion storage capability make GDY as an ideal ion-storage layer, which is crucial for the high performance of the EGT. This special ion-storage layer has never been reported by other materials, and thus we believe the introduction of GDY in EGTs might have better prospects in neuromorphic applications.

## 4. GDY-based memory devices

Memory is one of the most important components in the von Neumann computer. However, the explosive growth of data in the era of big data puts forward strict requirements for memory. Large data storage capability, high operation speed, ultralow energy consumption, and even multifunctional integration of optical sensing, data storage and processing are highly desirable [132–136]. 2D materials have become the most promising candidates for the development of novel memory devices. Compared with other 2D materials, GDY has its unique advantages in memory applications. For example, the inherent defects in GDY facilitate the storage of charges and thus a thinner dielectric layer can be used in the floating-gate memory, which can decrease the operation voltage for the programming and erasing operations. Moreover, the threshold-switching characteristics of graphdiyne oxide (GDYO) enable the direct injection of charges from control gate to floating gate. An ultrafast floating-gate memory can be developed using GDYO layer to replace the gate dielectric layer in conventional memory devices.

### 4.1 Optoelectronic memory

Optoelectronic memories that can perform programming and erasing operations under light pulses, offer great opportunities to integrate image sensing, data storage, and logic data processing [136,137]. In addition, the employment of optical inputs can broaden bandwidth and reduce electrical loss and delay of memory devices [37,120].

**Figure 7.**
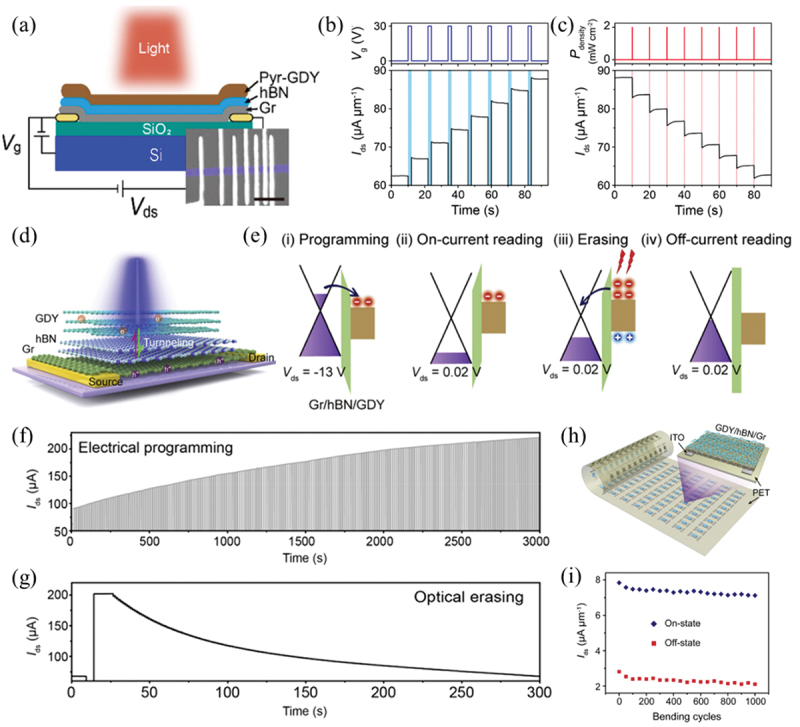
Optoelectronic multibit memories based on GDY van der Waals heterostructures. (a) Schematic illustration of the top-floating-gated optoelectronic memory based on the Pyr-GDY/hBN/graphene heterostructure. Inset is the false-colored scanning electron microscopy (SEM) image of the fabricated device with different channel lengths. Scale bar, 20 μm. (b) *I*_ds_−time curve under periodic gate voltage pulses (*V*_g_ = 30 V, *t* = 2 s), showing eight distinct storage states. (b) *I*_ds_−time curve under 450 nm light pulses (10 mW cm^−2^, 0.2 s) every 10 s, achieving nine memory levels. Reprinted from [107] with permission; © 2020, American Chemical Society. (d) Schematic illustration of a two-terminal floating-gate optoelectronic memory with a structure of GDY/hBN/graphene. (e) Schematic illustration of band diagrams for (I) programming, (II) on-current reading, (III) erasing and (IV) off-current reading. (f,g) *I*_ds_-time curves of the memory under periodic (f) *V*_ds_ pulses (−8 V, 1 s) and (g) optical pulses (450 nm, 10 mW cm^−2^, 100 ms), respectively, showing over 256 distinct current levels. (h) Schematic illustration of the memory fabricated on a flexible PET substrate. (i) Current variation of the device at the on and off states after successive bending cycles. Each cycle was performed at a bending radius of 10 mm for 5 s, followed by 5 s of waiting time. Currents were measured at *V*_ds_ = 0.1 V with an interval of 50 bending cycles. Reprinted from [100] with permission; © 2021, Elsevier Inc.

Floating-gate architecture is widely used in optoelectronic memories since photo-generated charges can be restricted in floating gate persistently [138,139]. The high density of states, large work function, direct bandgap and strong optical absorption endow GDY with the ability to store more charges and effectively suppress the gate-coupling ratio (GCR) degradation and cell-to-cell interference (CTCI), making it an ideal photoresponsive floating gate material. Chen and co-workers proposed a top-floating-gate optoelectronic memory with a structure of Pyr-GDY/hBN/graphene, in which Pyr-GDY and graphene acted as floating gate and channel, respectively (Figure 7(a)) [107]. As shown in Figure 7(b,c), this device could operate in electrical programming/electrical erasing and electrical programming/optical erasing modes, and more than eight distinct storage levels (3 bits) were demonstrated in both the electrical programming and optical erasing processes.

Subsequently, Chen and co-workers further proposed a two-terminal optoelectronic memory using a GDY/hBN/graphene vertical heterostructure (Figure 7(d)) [100]. Due to the absence of gate electrode and thus a thick and rigid blocking layer between gate and channel, this two-terminal floating-gate memory featured advantages of (i) an extremely short channel length facilitating miniaturization and integration of devices, (ii) excellent flexibility and stability and (iii) low operation voltage and energy consumption [140,141]. The mechanism of this device is illustrated in Figure 7(e). While applying a bias voltage pulse, a large potential drop between the drain and GDY is formed, driving electrons tunneling from the drain to the GDY floating gate and restricted in GDY. The optical erasing operation is achieved by applying an optical pulse, generating a large number of electron–hole pairs in GDY. Driven by the built-in electric field between GDY and graphene, photogenerated electrons tunnel back to graphene through the small triangular electron barrier of hBN, and photogenerated holes neutralize the trapped electrons in GDY. Benefiting from the large current difference between HRS and LRS, and long retention of over 10^5^ s, multibit storage over 256 levels (8 bits) had been demonstrated both in programming and erasing processes (Figure 7(f,g)). Moreover, a flexible device array was constructed on polyethylene terephthalate (PET) substrate (Figure 7(h)), which featured robust bending stability over 1000 bending cycles (Figure 7(i)), demonstrating its substantial potential in wearable electronics [142,143].

**Figure 8.**
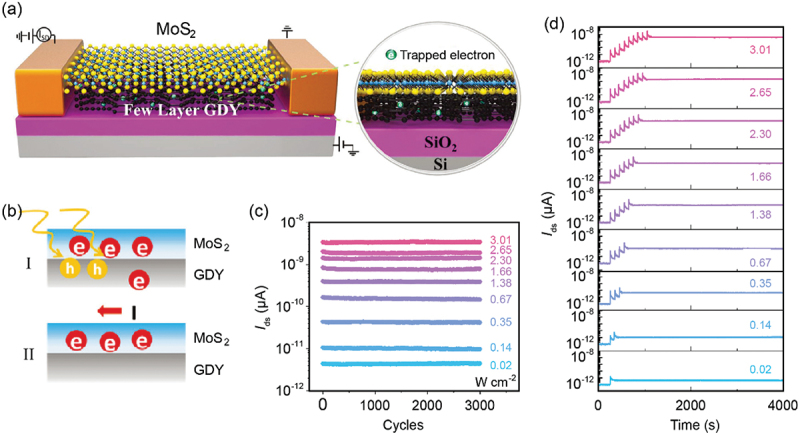
Direct-charge-trapping optoelectronic memory based on GDY van der Waals heterostructure. (a) Schematic illustration of the MoS_2_/GDY-based optoelectronic memory with direct-charge-trapping mechanism. (b) Schematic illustration of the memory for (I) the reset and (II) light programming processes. (c,d) (c) cyclic endurance and (d) retention characteristics of the memory in opto-electronic mode with light intensity ranging from 0.02 to 3.01 W cm^−2^. Reprinted from [58] with permission; © 2021 Wiley-VCH.

Direct charge trapping via interface, dangling bonds and defects is also widely used in optoelectronic memories [144–147]. Zhang and co-workers developed a direct-charge-trapping optoelectronic memory based on MoS_2_/GDY heterostructure [58]. The device structure is illustrated in Figure 8(a), in which MoS_2_ was used as the channel and oxygen-plasma-treated GDY served as the charge-trapping layer. While applying a positive gate voltage (10 V) to the device, electrons in the channel were injected into GDY. The oxygen-plasma-treated GDY contained numerous oxygen-containing groups, which can act as localized charge-trapping sites, and the injected electrons were trapped in these sites even after removing the gate voltage. The trapped electrons in GDY induced a negative electric field to MoS_2_ channel, resulting in the depletion of free carriers in MoS_2_. Thus, the device was tuned to an off state, and this process was defined as the ‘reset’. A negative *V*_g_ pulse can be used to extract the trapped electrons back to MoS_2_, and the device returned back to an on state. By controlling the amplitude of the applied *V*_g_ voltage (−10 V to −80 V), multibit storage was demonstrated. Optical induced release of trapped electrons can also be achieved by applying a 532 nm laser pulse. Photogenerated holes in MoS_2_ combined with the trapped electrons in GDY, leaving photogenerated electrons in MoS_2_, which can be regarded as the release of the trapped electrons from GDY to MoS_2_ (Figure 8(b)). Nine distinct storage levels with long retention time (>4000 s) and excellent cyclic stability (>3000 cycles) were achieved by adjusting the intensity of light pulse from 0.02 W cm^−2^ to 3.01 W cm^−2^ (Figure 8(c,d)). The excellent charge-trapping capability of GDY is the footstone of this direct-charge-trapping optoelectronic memory.

### 4.2 High-speed nonvolatile memory

The increasing gap of data-processing speed between memories and processors, known as ‘memory wall’, is becoming one of the most severe challenges in von Neumann computers. Memory with high operation speed, long retention and low power is highly desirable in next-generation electronics. Great efforts have been made to develop novel ultrafast nonvolatile memories in the past decade. Especially, the atomically sharp interfaces of 2D van der Waals heterostructures make it possible to develop high-speed floating gate memories [14,15].

**Figure 9.**
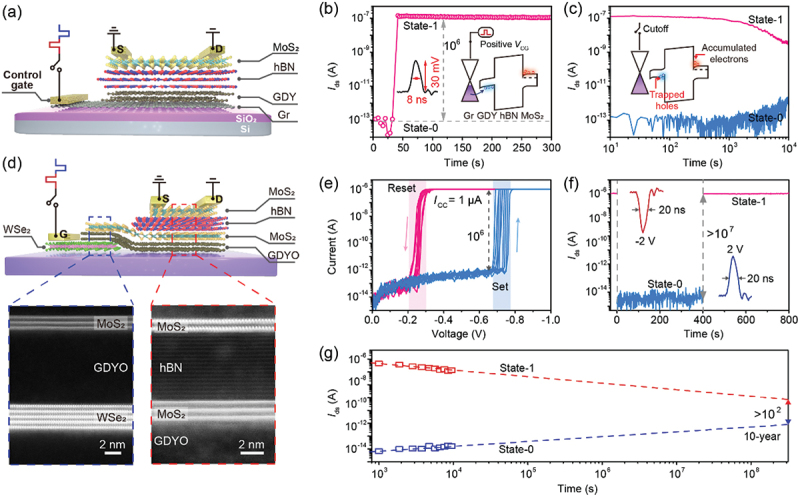
Ultrafast nonvolatile memories based on GDY van der Waals heterostructures. (a) Schematic illustration of a direct-charge-injection floating-gate memory with a structure of MoS_2_/hBN/GDY/graphene. (b) High-speed writing operation achieved by applying a 30 mV/8 ns *V*_cg_ pulse to control gate. Insets: the real waveform of *V*_cg_ pulse (left) and band diagrams for the writing operation (right). (c) Retention characteristics of the memory at state-0 and state-1. Inset: the band diagrams of the memory at state-1. Reprinted from [108] with permission; © 2021 Wiley-VCH. (d) Schematic illustration of an ultrafast floating-gate memory based on MoS_2_/hBN/MoS_2_/GDYO/WSe_2_ heterostructure (upper panel) and aberration-corrected scanning transmission electron microscopy (STEM) images of cross sections of MoS_2_/GDYO/WSe_2_ (left in lower panel) and MoS_2_/hBN/MoS_2_/GDYO (right in lower panel) heterostructures. (e) *I*–*V* curves of the GDYO-based TS device sweeping from 0 V to −1 V and back to 0 V. (f) High-speed writing and erasing operations achieved by applying a −2 V/20 ns and **+**2 V/20 ns *V*_cg_ pulses, respectively. Insets: the real waveform of *V*_cg_ pulses. (g) Retention characteristics of the memory at state-1 and state-0 with a linear extrapolation to 10 years. Reprinted from [109] with permission; © 2022, Springer Nature Ltd.

In 2018, Zhou and co-workers proposed a direct-charge-injection strategy instead of Fowler-Nordheim (FN) tunneling mechanism to achieve a nanosecond operation speed [13]. However, the poor charge trapping capability of the floating gate lead to a limited retention characteristic (10 s). As discussed above, GDY features excellent charge-trapping capability that can trap charges for quite a long time. Chen and co-workers developed a direct-charge-injection floating-gate memory with a structure of MoS_2_/hBN/GDY/graphene (Figure 9(a)), in which GDY and graphene acted as the charge-trapping layer and control gate, respectively [108]. The absence of the blocking layer between charge-trapping layer and control gate enabled the direct injection of holes from graphene to GDY while applying a positive *V*_cg_ pulse to the control gate. Benefiting from this direct-charge-injection strategy, the writing operation of the memory was achieved by applying a nanosecond ultralow *V*_cg_ pulse (30 mV, 8 ns), with an on/off ratio of 10^6^ (Figure 9(b)). The injected holes were restricted in GDY by the charge-trapping sites, as well as the downward band bending at GDY/graphene interface, resulting in a long retention time over 10^4^ s (Figure 9(c)). However, compared with the hole-trapping process, the detrapping of holes had a larger energy barrier induced by the numerous alkyne bonds of GDY [58]. Together with the asymmetric band bending at GDY/graphene interface, the extraction of trapped holes from GDY required a relatively larger voltage and longer duration (−500 mV, 100 μs) for erasing operation.

In order to obtain symmetric writing/erasing speed in nanosecond timescale, Chen and co-workers further modified the architecture of the floating-gate memory as illustrated in Figure 9(d) [109]. Distinct from conventional floating-gate memory, this device utilized a threshold-switching (TS) layer, i.e. GDYO, rather than a blocking layer to connect the floating gate (MoS_2_) and control gate (WSe_2_). The GDYO TS layer fabricated by UV-ozone treatment contained numerous oxygen-containing groups, and its initial state was dielectric (off state). While applying a bias, oxygen-containing groups in GDYO migrated, forming GDY conductive filaments (CFs). Due to the large-pore structure of GDY, oxygen-containing groups diffused back to its initial region after removing the bias, leading to the rupture of GDY CFs. Thus, the GDYO layer featured a TS characteristic with switching time of approximately 20 ns (Figure 9(e)). Therefore, while applying a negative *V*_cg_ pulse to the control gate, the GDYO TS layer switched to on state within 20 ns, and electrons can be directly injected into the floating gate through GDYO. After the writing pulse, GDYO TS layer spontaneously switched to off state, restricting the injected electrons into the floating gate. The erasing operation was achieved by applying a positive *V*_cg_ pulse, which can switch GDYO TS layer on and extract the injected electrons through GDYO. As shown in Figure 9(f,g), this memory demonstrated a nanosecond writing/erasing speed (20 ns) and ultralong retention time (10 years) with an on/off ration of 10^7^. In particular, this device featured a quite low operation voltage (2 V), which was over one order of magnitude lower than that of conventional floating-gate memories [14,15], significantly decreasing energy consumption (10 fJ) and improving complementary-metal-oxide-semiconductor (CMOS) compatibility. In addition, by controlling the amplitude of *V*_cg_ pulse and thus the injected electrons, the device realized a 3-bit storage with a few nanoseconds, which was a great progress compared with the slow operation speed of previously reported multilevel memories [100,105,137,138,148].

## 5. Summary and perspectives

In this review, we summarize recent research progress in GDY-based neuromorphic devices and memories, including their working mechanisms and applications. As a new 2D carbon allotrope, GDY features highly π-conjugated structure, evenly distributed pores, and abundant charge-trapping sites, making it especially suitable for neuromorphic and memory devices. In recent years, some novel synaptic devices and memory devices have been developed based on GDY, such as optoelectronic synapses, electrolyte-gate synaptic transistors, optoelectronic multibit memories, high-speed nonvolatile memories and memristors. In these devices, GDY acts as the key components, such as photoresponsive charge trapping layer, high-speed threshold-switching layer, and ion-storage layer, which are essential for the high performance of the devices. Based on these GDY-based devices with different structures, various applications such as neuromorphic computing, artificial visual systems, nociceptive signal processing system, associative learning, and logic-in-memory have been demonstrated, which exhibit unique advantages in these applications. For example, the GDY-based EGT has an ultralow energy consumption, linear conductance-update characteristic, and robust stability, which can significantly improve the accuracy and energy-efficiency of ANN for neuromorphic computing. In addition, the light-controlled bidirectional synaptic response enables the construction of two-terminal synapses with simple architecture, which facilitates the development of flexible artificial visual system for wearable applications. And the optically controlled dual-mode memristors can simplify the fabrication and integration of fully memristive neuromorphic hardware, such as SNNs for classification and nociceptive signal processing systems.

However, there are still many challenges in the development of GDY-based devices and their practical applications. Although GDY films suitable for device application have been prepared, these films are polycrystalline containing numerous defects and grain boundaries, which significantly degrade the performance of the as-prepared GDY films. It is still a great challenge to synthesize single-crystal monolayer GDY films thus far, and some theoretically predicted superior properties, for example, the ultrahigh carrier mobility at room temperature, are not yet achieved. As a result, electrical devices using GDY as channel material always have a poor performance since charge transport in GDY is seriously suppressed by defects and grain boundaries. GDY in the existing devices usually serves as a function layer.

The major challenges for the synthesis of high-quality or even single-crystal GDY lie in the facts of (i) the free rotation around C – C bonds in monomers which would lead to a highly branched or cross-linked framework with less ordering and (ii) the limited step-down diffusion of monomers adsorbed on the growth substrates which would lead to the accumulation and nucleation of admolecules and out-of-plane growth [99]. Fortunately, breakthroughs and innovations are achieved constantly with the great efforts of researchers in this field. Aiming at single-crystal GDY, new synthetic methodologies even beyond solution-phase coupling reaction, for example, chemical vapor deposition (CVD) [149], will be developed in the future, which can bridge the gap between ‘real GDY’ and ‘ideal GDY’. Through CVD technique, GDY monomer molecules can be deposited on the surface of silver foil in an ordered and quantitative manner (similar to the preparation of graphene), and the number of defects and grain boundaries will be significantly reduced, facilitating the preparation of high-quality ultrathin GDY films.

Besides the synthesis methods, other challenges for the large-scale application of GDY in memory and neuromorphic computing are the inconsistent device performance induced by the poorly controlled defect density, and the compatibility of fabrication techniques with conventional semiconductor technologies. Defect engineering might be used to control the defect density between different batches more precisely, and a suitable programming method, such as a ‘closed-loop’ method [150], can be used to circumvent the device-to-device variation (DDV) for large-scale device arrays. In addition, with the development of techniques for large-scale integrated circuits based on 2D materials [66], GDY-based device arrays and neuromorphic systems can be manufactured conveniently.

Anyway, huge progress in GDY-based novel memory and neuromorphic devices has been made in recent years, and the unique structure and properties of GDY solve some major challenges in these fields. GDY has demonstrated its promising potential in neuromorphic applications. In the future, we will pay more efforts on the large-scale preparation of GDY films and fabrication of GDY-based device arrays, to promote the practical applications of GDY.
